# p21^Waf1/Cip1^ Is a Novel Downstream Target of 40S Ribosomal S6 Kinase 2

**DOI:** 10.3390/cancers16223783

**Published:** 2024-11-10

**Authors:** Alakananda Basu, Zhenyu Xuan

**Affiliations:** 1 Department of Microbiology, Immunology and Genetics, University of North Texas Health Science Center, Fort Worth, TX 76107, USA; 2 Department of Biological Sciences, Center for Systems Biology, University of Texas at Dallas, Richardson, TX 75080, USA; zhenyu.xuan@utdallas.edu

**Keywords:** S6K2/*RPS6KB2*, S6K1, p21/*CDKN1A*, cJun/*JUN*, Akt1, Akt2, JNK, apoptosis, chemoresistance, breast cancer

## Abstract

**Background/Objectives**: The ribosomal S6 kinase 2 (S6K2) acts downstream of the mechanistic target of rapamycin complex 1 and is a homolog of S6K1 but little is known about its downstream effectors. The objective of this study was to use an unbiased transcriptome profiling to uncover how S6K2 promotes breast cancer cell survival. **Methods**: RNA-Seq analysis was performed to identify novel S6K2 targets. Cells were transfected with siRNAs or plasmids containing genes of interest. Western blot analyses were performed to quantify total and phosphorylated proteins. Apoptosis was monitored by treating cells with different concentrations of doxorubicin. **Results**: Silencing of S6K2, but not S6K1, decreased p21 in MCF-7 and T47D breast cancer cells. Knockdown of Akt1 but not Akt2 decreased p21 in MCF-7 cells whereas both Akt1 and Akt2 knockdown attenuated p21 in T47D cells. While Akt1 overexpression enhanced p21 and partially reversed the effect of S6K2 deficiency on p21 downregulation in MCF-7 cells, it had little effect in T47D cells. S6K2 knockdown increased *JUN* mRNA and knockdown of cJun enhanced p21. Low concentrations of doxorubicin increased, and high concentrations decreased p21 levels in T47D cells. Silencing of S6K2 or p21 sensitized T47D cells to doxorubicin via c-Jun N-terminal kinase (JNK)-mediated downregulation of Mcl-1. **Conclusions**: S6K2 knockdown enhanced doxorubicin-induced apoptosis by downregulating the cell cycle inhibitor p21 and the anti-apoptotic protein Mcl-1 via Akt and/or JNK.

## Simple Summary

The majority of breast cancers express estrogen receptors (ER) and are treated with antiestrogens. However, many patients relapse and do not respond to further treatment. Therefore, an understanding of which molecules/pathways are affected in breast cancer is important. The Akt/mTOR (mechanistic target of rapamycin) pathway is often altered in breast cancers and is an important target for cancer therapy. The ribosomal S6 kinase 2 (S6K2) acts downstream of mTOR and has been associated with ER-positive breast cancers, but little is known about how S6K2 functions in these cancers. The aim of our present study was to identify potential downstream effectors of S6K2. We identified p21/*CDKN1A* as a novel downstream target of S6K2 in ER-positive breast cancer cells and showed that S6K2 acts via Akt and JNK signaling pathways to regulate p21 and an upregulation of p21 contributes to chemoresistance. Thus, targeting components of the S6K2/p21 signaling pathway could reverse chemoresistance.

## 1. Introduction

The mechanistic target of rapamycin (mTOR) acts downstream of the phosphatidylinositol-3-kinase (PI3K)/Akt/mTOR pathway and plays a central role in breast cancer [1]. It forms two complexes: mTOR complex 1 (mTORC1) and mTOR complex 2 (mTORC2) [2]. The ribosomal S6 kinase 2 (S6K2) acts downstream of mTORC1 and is a homolog of p70 S6 kinase or S6K1 [3,4,5]. Both homologs phosphorylate ribosomal protein S6, but they exhibit redundant, distinct, or even opposite functions [6]. While numerous studies have focused on S6K1, much less is known about S6K2.

Breast cancer is the second leading cause of cancer-related death in women in the United States (www.cancer.org, accessed on 25 March 2024). There are four major types of breast cancer: Estrogen receptor (ER) positive (luminal A and luminal B), HER-2 enriched and basal-like (often used synonymously with triple-negative) [7]. The majority of breast cancer patients express estrogen receptor (ER)-α [8].

Several studies implicated S6K2 in ERα signaling. S6K2, encoded by *RPS6KB2*, is localized on chromosome 11q13 [9,10], which constitutes a high-risk subgroup of ER-positive breast cancers [11]. *RPS6KB2* gains/amplifications have been associated with poor prognosis and resistance to endocrine therapy [9,10]. Based on our analysis of the breast cancer study (BRCA) of The Cancer Genome Atlas (TCGA), S6K2 is overexpressed in breast cancer [12]. 

In the present study, we took an unbiased approach to identifying downstream targets of S6K2 in ER-positive breast cancers. We made a novel observation that p21 encoded by *CDKN1A* is a downstream target of S6K2, which regulates p21 via Akt-dependent and -independent pathways. We also showed that S6K2 negatively regulates cJun and the knockdown of cJun increases p21 level. Finally, the knockdown of S6K2 enhances chemosensitivity by downregulating both the cell cycle inhibitor p21 and the antiapoptotic protein Mcl-1.

## 2. Materials and Methods

### 2.1. Materials

A goat polyclonal antibody against S6K2 was purchased from R&D Systems (Minneapolis, MN, USA). Rabbit monoclonal antibodies against S6K1, Akt1, Akt2, phospho-Akt1 (Ser473), phospho-Akt2 (Ser474), phospho-S6 (Ser240/244), cJun, phospho-SAPK/JNK, Mcl-1, phospho-CDK2 (T160), α-actinin, PARP, cleaved caspase-7, p21, and a monoclonal antibody against JNK1 were obtained from Cell Signaling Technology (Danvers, MA, USA). A mouse monoclonal antibody against GAPDH was obtained from Santa Cruz Biotechnology (Dallas, TX, USA). Mouse monoclonal antibodies against actin and tubulin were purchased from Sigma-Aldrich (St Louis, MO, USA). Doxorubicin was obtained from LC Laboratories (Woburn, MA, USA). Horseradish peroxidase-conjugated donkey anti-rabbit, goat anti-mouse, and mouse anti-goat antibodies were obtained from Jackson ImmunoResearch Lab. Inc. (West Grove, PA, USA). Polyvinylidene difluoride membrane, *JUN* siRNAs and lipofectamine 3000 were from Thermo Fisher Scientific (Waltham, MA, USA) and Millipore (Bedford, MA, USA). An enhanced chemiluminescence detection kit was from Perkin-Elmer (Shelton, CT, USA) and Thermo Fisher Scientific (Waltham, MA, USA). Control non-targeting and target-specific siRNAs were obtained from GE Dharmacon (Chicago, IL, USA). A lipofectamine RNAiMax transfection reagent was obtained from Invitrogen, (Carlsbad, CA, USA). Protease inhibitor and phosphatase inhibitor cocktails were purchased from Calbiochem/EMD-Millipore (Bedford, MA, USA). 

### 2.2. Cell Culture and Transfection

T47D and MCF-7 cells originally obtained from ATCC were cultured in RPMI 1640 medium supplemented with 8% fetal bovine serum and 2 mM glutamine and were kept in a humidified incubator at 37 °C with 95% air and 5% CO_2_. siRNA transfections were performed with 10 nM control non-targeting or target-specific siRNAs using a Lipofectamine^®^ RNAiMAX transfection reagent (Invitrogen, Carlsbad, CA, USA) and incubated for 48 h to 72 h. Unless otherwise mentioned, SMARTpool siRNA, which is a combination of four individual siRNAs, was used. The concentration of each individual siRNA in the SMARTpool siRNA was 2.5 nM for a total of 10 nM final concentration. The extent of transfection was determined by a Western blot analysis. For Akt overexpression studies, cells transfected with S6K2 siRNA were incubated for 24 h and then infected with or without an adenoviral vector containing Akt. MCF-7 cells were transfected with pcDNA3 or pcDNA3 containing HA-tagged S6K2 construct [5] obtained from Addgene using lipofectamine 2000 and the manufacturer’s protocol. 

### 2.3. RNA Isolation, NGS Sequencing, and Real-Time Quantitative PCR

The total RNA was extracted from T47D cells transfected with control non-targeting and S6K2 siRNA using the RNeasy kit (Qiagen, Germantown, MD, USA) and the manufacturer’s protocol. The purity of the RNA was determined, and high-quality RNA samples (RQ1 9.8–10) were submitted to the University of Southwestern’s genomic core facility for library preparation and sequencing. 

A reverse transcription was performed using SuperScript IV First-Strand Synthesis System (Invitrogen 18091050) and the manufacturer’s protocol. A real-time PCR was performed using TaqMan Gene Expression assays using a primer and probe for *RPS6KB2* (Hs00177689_m1) and *CDKN1A* (Hs00355782_m1) (Invitrogen, Waltham, MA, USA). Duplicate samples were run using a Applied Biosystems StepOne Real-Time PCR System and data were analyzed as described in the manual. All quantifications were normalized with *GAPDH* (Hs03929097_g1) and *18SRNA* (Hs99999901_s1) to account for variability in the initial concentration, quality of RNA, and the conversion efficiency of the reverse transcription reaction. It was calculated with the ΔΔCt method and compared to the control siRNA. 

### 2.4. Western Blot Analysis

Cells were lysed in extraction buffer containing 20 mM Tris-HCl, pH 7.4, 0.15 M NaCl, 1 mM EGTA, 1 mM EDTA, 1.0% Nonidet-40, 10 mM β-glycerophosphate, protease inhibitor cocktail, and phosphatase inhibitor cocktail. Equivalent amounts of total proteins (5–25 μg) were electrophoresed by SDS-PAGE and transferred electrophoretically to a polyvinylidene difluoride membrane. The blots were visualized using the enhanced chemiluminescence detection reagents and the manufacturer’s protocol. The blots were probed with actin, actinin, GAPDH, or tubulin to control equal loading.

### 2.5. Statistical Analyses

The intensities of immunoreactive proteins were quantified using ImageJ (version 2.14.0/1.5f) software (National Institutes of Health, Bethesda, MD, USA). The statistical significance was determined by student’s paired *t*-test using GraphPad Prism software. A *p*-value of <0.05 was considered statistically significant.

### 2.6. RNA-Seq Data Analyses

We used Tophat [13] to map all RNA-seq reads with human reference genome and a transcriptome of build Hg38. We used RSEM [14] to quantify gene expression and DESeq [15] to identify differentially expressed genes (DEGs). We also used Cufflink/Cuffdiff [16] packages to identify DEGs. The shared DEGs from both analyses were used for functional enrichment analysis by DAVID [17] (https://david.ncifcrf.gov/, accessed on 3 March 2024). The mostly enriched functional annotation clusters with at least one gene ontology [18] term significantly enriched (FDR < 0.05) were selected, and the representative term of each cluster was reported.

## 3. Results

### 3.1. Transcriptome Profiling of Breast Cancer Cells Following Knockdown of S6K2

**Figure 1:**
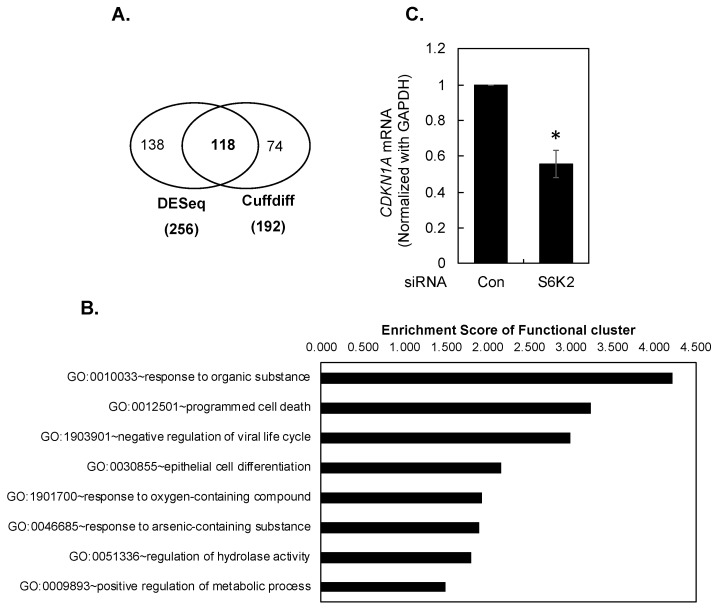
RNA-Seq analysis. (**A**). Venn graph of DEGs detected using both Cuffdiff and DESeq following S6K2 KD. FDR < 0.05 was applied for each method. (**B**). The representative gene ontology terms of functional annotation clusters, which are significantly enriched in 118 shared DEGs (FDR < 0.05). (**C**). Densitometric quantification of *CDKN1A* mRNA normalized with GAPDH control. The asterisk (*) indicates a significant difference from control siRNA-transfected cells (*p* < 0.05) using paired Student’s *t*-test.

Since little is known about S6K2 signaling, we took an unbiased approach to identify potential downstream target(s) of S6K2. We transfected T47D breast cancer cells with S6K2 siRNA or control non-targeting siRNA and focused on those differentially expressed genes (DEG) that were increased more than 2-fold using both DESeq [15] and Cuffdiff [16] and a false discovery rate (FDR) of less than 0.05. We identified 118 significantly differentially expressed genes, of which 70 genes were upregulated more than 2-fold and 48 genes were downregulated more than 2-fold (Figure 1A and Supplementary Table S1). Using DAVID (https://david.ncifcrf.gov/) [17], we found eight clusters of gene ontology terms in biological process and molecular functions with at least one significantly enriched term in each cluster (Figure 1B and Supplementary Table S2). These include “response to organic substance”, “programmed cell death”, “negative regulation of viral life cycle”, and “epithelial cell differentiation”. Among all these different biological processes, *CDKN1A* is the most shared gene. We therefore decided to focus on *CDKN1A* for further study. *CDKN1A* was decreased 2.6-fold by S6K2 knockdown using both Cuffdiff (*p* value = 5.0 × 10^−5^) and DESeq (*p* value = 1.09 × 10^−81^). We also validated the effects of S6K2 KD on *CDKN1A* expression by RT-qPCR. As shown in Figure 1C, knockdown of S6K2 caused an approximately 40% decrease in *CDKN1A* mRNA.

### 3.2. Differential Effects of S6K1 and S6K2 on p21 Protein

**Figure 2:**
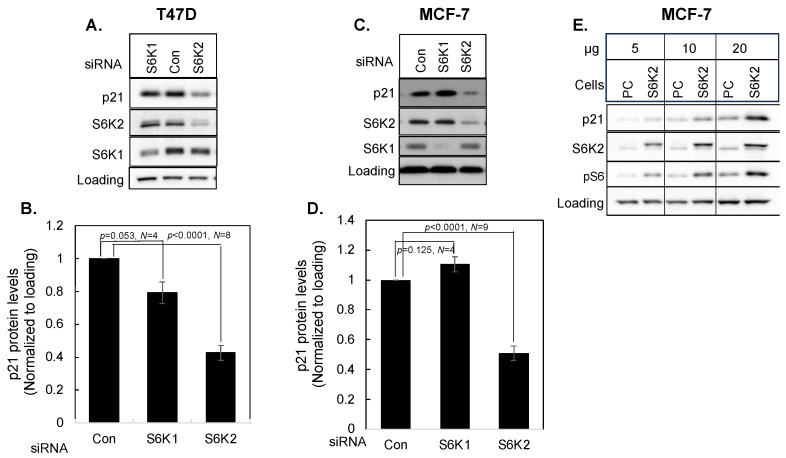
T47D (**A**,**B**) or MCF-7 (**C**,**D**) cells were transfected with or without control non-targeting siRNA or SMARTpool (SP) S6K1 or S6K2 siRNA. Western blot analyses were performed with indicated antibodies. The intensity of p21 was determined using ImageJ and normalized with respect to loading control. Each bar represents mean ± S.E. *p* values were calculated using a paired Student’s *t* test. (**E**). Different concentrations of cell lysates from MCF-7 cells transfected with an empty vector pcDNA3 (PC) or a vector containing S6K2 construct were subjected to Western blot analyses with indicated antibodies.

We performed a Western blot analysis to determine the effect of S6K2 knockdown on the expression of *CDKN1A* gene product p21 and extended our study to another ER-positive breast cancer cell line. As shown in Figure 2, knockdown of S6K2 caused a substantial decrease in p21 protein levels in both T47D and MCF-7 cells. Since S6K homologs share some functions, but may also exhibit distinct functions [6], we also determined if the silencing of S6K2 homolog S6K1 affects p21 expression. While knockdown of S6K2 caused a 2.34-fold decrease in p21 in T47D cells, S6K1 knockdown had only a modest effect (Figure 2A,B). In MCF-7 cells, S6K2 knockdown caused a 2-fold decrease in p21 (Figure 2D) whereas knockdown of S6K1 had little effect on p21 (Figure 2C,D). In fact, in some experiments, S6K1 KD appears to cause an increase in p21, but based on several independent experiments the effect was not statistically significant. To further corroborate the regulation of p21 by S6K2, we examined the effect of S6K2 overexpression on p21 level. Figure 2E shows that overexpression of S6K2 in MCF-7 cells caused a concentration-dependent increase in p21 and this was associated with an increase in phosphorylation of ribosomal S6, a substrate of S6K kinases. 

**Figure 3:**
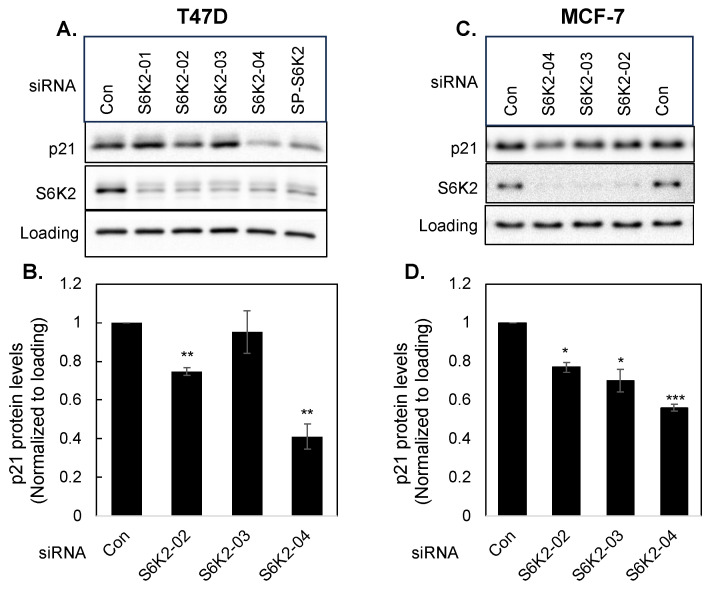
T47D (**A**,**B**) or MCF-7 (**C**,**D**) cells were transfected with indicated siRNAs and Western blot analyses were performed with indicated antibodies. Each bar represents the mean ± S.E of four independent experiments. *p* values were calculated using paired Student’s *t* test of control versus individual siRNA as described under Figure 2. ***, *p* ≤ 0.0005; **, *p* ≤ 0.005; *, *p* ≤ 0.05.

We then compared the effects of four different S6K2 siRNAs with control non-targeting siRNA on p21 levels in both T47D (Figure 3A,B) and MCF-7 (Figure 3C,D) cells. S6K2 siRNA-04 was most effective in reducing p21 in both MCF-7 and T47D cells and its effect was similar to SMARTpool siRNA (Figure 2). Unless otherwise mentioned, we used SMARTpool S6K2 siRNA in subsequent experiments.

### 3.3. Effects of Akt1 vs. Akt2 on p21 Levels

**Figure 4:**
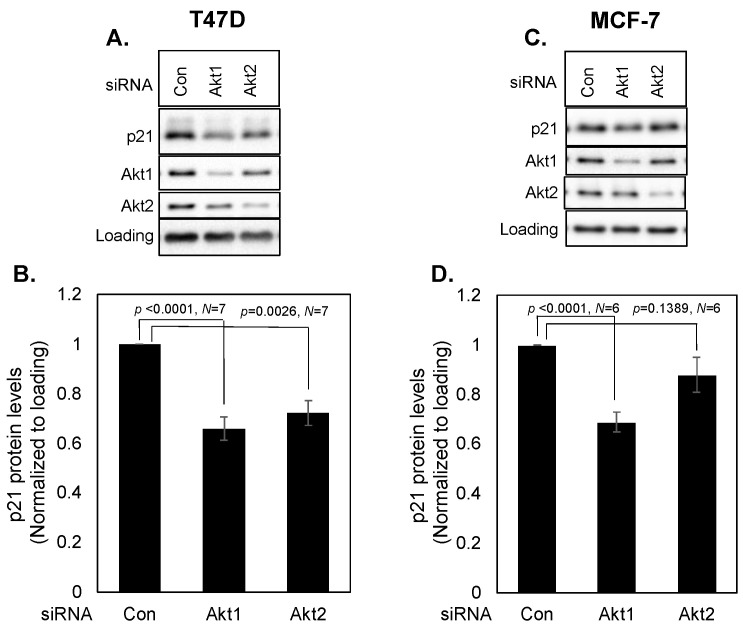
T47D (**A**,**B**) or MCF-7 (**C**,**D**) cells were transfected with indicated siRNAs and Western blot analyses were performed with indicated antibodies. Each bar represents mean ± S.E of at least six independent experiments. *p* values were calculated using paired Student’s *t* test.

We have previously shown that S6K2 acts upstream of the Akt signaling pathway in MCF-7 cells [19]. We therefore examined if S6K2 regulates p21 via Akt. We also compared the effects of Akt1 versus Akt2 siRNA on p21 levels. Figure 4 shows that knockdown of Akt1 caused a more than 30% decrease in p21 levels in both MCF-7 and T47D cells. While knockdown of Akt2 also decreased p21 in T47D cells albeit less efficiently than Akt1 knockdown (Figure 4A,B), Akt2 knockdown had little effect in MCF-7 cells (Figure 4C,D).

**Figure 5:**
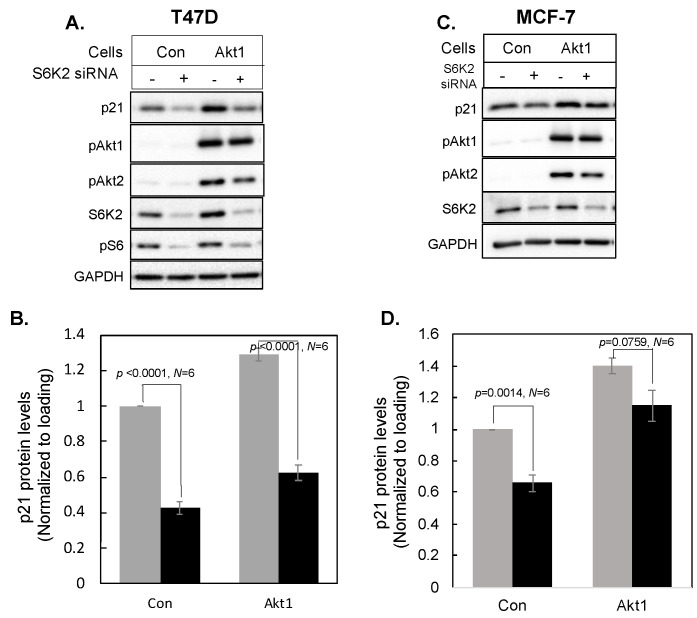
T47D (**A**,**B**) or MCF-7 (**C**,**D**) cells were transfected with control or S6K2 siRNA and then infected with or without adenoviral vectors containing Akt1. Western blot analyses were performed with indicated antibodies. Each bar represents mean ± S.E of six independent experiments. *p* values calculated using paired *t* test of control versus Akt1 overexpressing cells: T47D, *p* = 0.0007; MCF-7, *p* = 0.0005; Light gray bar, control siRNA; black bar, S6K2 siRNA.

To determine if S6K2 regulates p21 via the Akt signaling pathway, we examined if overexpression of Akt1 could reverse the decrease in p21 caused by S6K2 depletion. Figure 5 shows that adenoviral vector-mediated delivery of Akt1 in MCF-7 or T47D cells increased phosphorylation of both Akt1 and Akt2, indicating activation of both isoforms, whereas S6K2 knockdown attenuated the increase in phosphorylation of Akt1 and Akt2. 

Akt1 overexpression caused an increase in p21 in both T47D (Figure 5A) and MCF-7 (Figure 5C) cells. While the overexpression of Akt1 partially reversed the effect of S6K2 knockdown on the decrease in p21 in MCF-7 cells (Figure 5D), Akt1 overexpression failed to rescue the effect of S6K2 knockdown in T47D cells (Figure 5B). These results suggest that S6K2 may regulate p21 via both Akt-dependent and independent pathways.

### 3.4. Effect of cJun on p21

**Figure 6:**
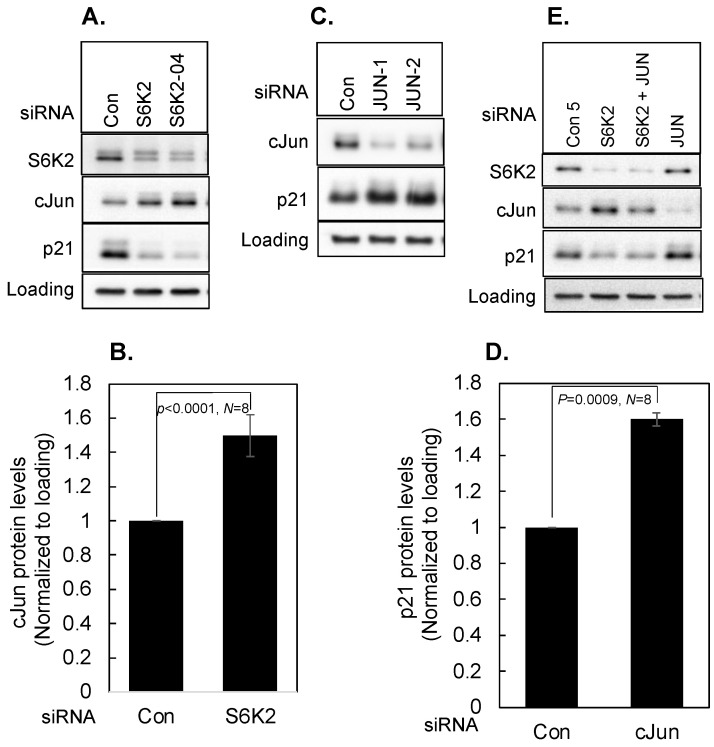
T47D cells were transfected with indicated siRNAs. Western blot analyses were performed with indicated antibodies (**A**,**C**,**E**). The intensities of cJun (**B**) and p21 (**D**) were determined using ImageJ and normalized with respect to loading controls. Each bar represents mean ± S.E. *p* values were calculated using paired Student’s *t* test.

We have previously shown that S6K2 regulates apoptosis via c-Jun N-terminal kinase (JNK) independent of the Akt signaling pathway in T47D breast cancer cells [20]. Based on our RNA-Seq data, knockdown of S6K2 enhanced *JUN* mRNA by 2-fold (Supplementary Table S1). It was one of the most shared DEGs among the enriched functional clusters, including programmed cell death (Figure 1B and Supplementary Table S2). Western blot analysis revealed that a decrease in p21 by S6K2 knockdown was associated with an increase in cJun protein (Figure 6A,B). To determine if an increase in cJun by S6K2 knockdown was responsible for the decrease in p21, we compared the effects on two different cJun siRNAs on p21 levels. Figure 6B shows that both siRNAs increased p21 levels (Figure 6C,D) and the silencing of S6K2 attenuated the increase in p21 caused by cJun knockdown (Figure 6E). These results suggest that S6K2 negatively regulates cJun, and an increase in cJun by S6K2 knockdown is responsible for the downregulation of p21 in T47D cells. 

### 3.5. Effect of S6K2, p21 and cJun on Chemosensitivity

**Figure 7:**
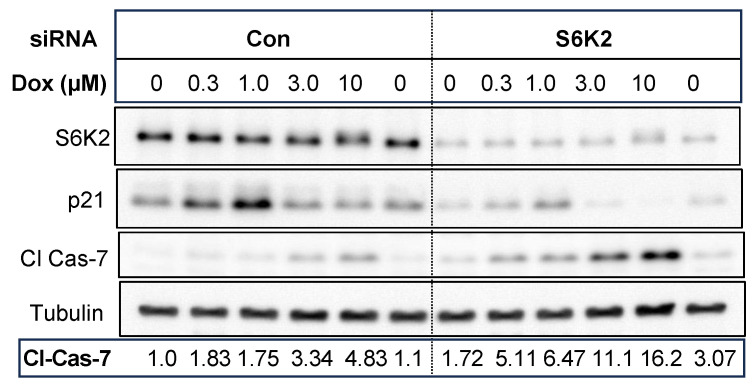
T47D cells were transfected with control non-targeting siRNA or S6K2 siRNA and then treated with indicated concentrations of doxorubicin (Dox). Western blot analyses were performed with indicated antibodies. The band corresponding to cleaved caspase-7 was quantified using ImageJ and the intensities of bands normalized with loading controls are shown.

To determine if a decrease in p21 by S6K2 knockdown was associated with increased sensitivity to an apoptotic stimulus [19,20], we treated T47D cells with different concentrations of the chemotherapeutic drug doxorubicin. Figure 7 shows that treatment of T47D cells with low concentrations of doxorubicin induced p21 up to 1 µM doxorubicin followed by a decrease in p21. During apoptosis, procaspases are cleaved to generate active caspases, and the activation of effector caspases ultimately cleaves critical cellular proteins, such as poly-ADP ribose polymerase (PARP). As shown in Figure 7, low concentrations of doxorubicin had little effect on the processing of effector caspase-7 in control siRNA transfected cell. The generation of cleaved caspase (Cl cas)-7 increased at concentrations of doxorubicin that also caused downregulation of p21. S6K2 knockdown decreased both basal and doxorubicin-induced p21 and enhanced processing of procaspase-7. These results suggest that knockdown of S6K2 enhanced cellular sensitivity to doxorubicin by lowering p21 levels.

**Figure 8:**
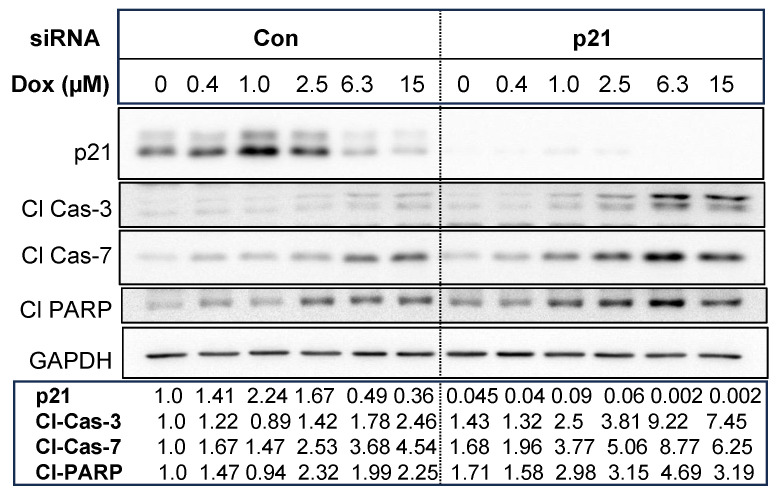
T47D cells were transfected with control non-targeting siRNA or p21 siRNA and then treated with indicated concentrations of doxorubicin. Western blot analyses were performed with indicated antibodies. The bands corresponding to p21, cleaved caspase-3, caspase-7, and PARP were quantified using ImageJ, and the intensities of bands normalized with loading controls are shown.

We then examined if silencing of p21 enhances chemosensitivity. As shown in Figure 8, knockdown of p21 enhanced processing of caspase-3, caspase-7, and PARP. The generation of cleaved fragments of effector caspases appeared at 1 µM doxorubicin and increased with higher doxorubicin concentrations. These results suggest the depletion of p21 enhanced cellular sensitivity to doxorubicin.

**Figure 9:**
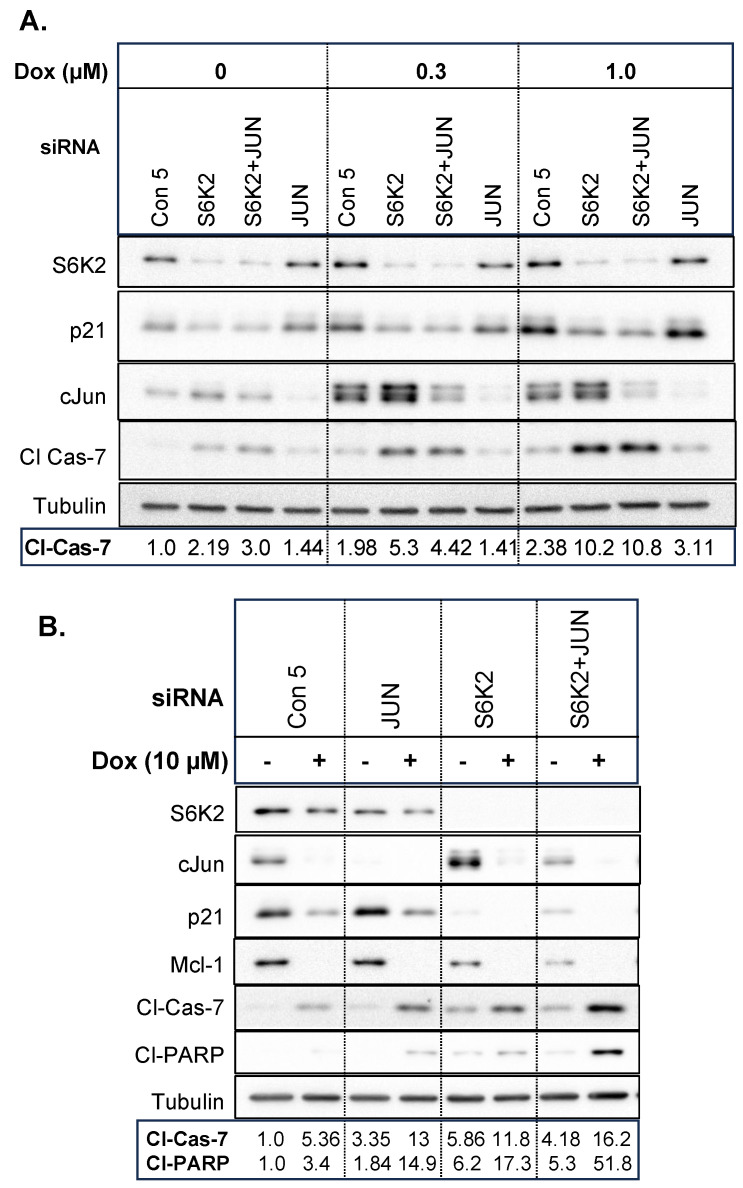
T47D cells were transfected with control non-targeting siRNA, S6K2 and/or c-Jun siRNA and then treated with or without 0.3 and 1.0 µM (**A**) or 10 µM (**B**) doxorubicin. Western blot analysis was performed with indicated antibodies. The band corresponding to cleaved caspase-7 or PARP was quantified using ImageJ and the intensities of bands were normalized with tubulin.

Since S6K2 knockdown decreased p21, whereas cJun knockdown enhanced p21 level, we examined if knockdown of cJun inhibits the S6K2 knockdown-induced increase in apoptotic markers. Figure 9A shows that 0.3 µM doxorubicin increased cJun and knockdown of S6K2 further increased cJun and decreased p21 levels. Subsequent treatment with doxorubicin gradually decreased cJun level such that it was undetectable at 10 µM doxorubicin (Figure 9B). While cJun knockdown had little effect on the cleavage of procaspase-7 at low concentrations of doxorubicin (Figure 9A), it enhanced cleaved caspase-7 at 10 µM doxorubicin (Figure 9B). The combined knockdown of S6K2 and cJun had little or no effect on cleaved caspase-7 at low concentrations of doxorubicin (Figure 9A) but appears to enhance the cleavage of procaspase-7 and PARP at 10 µM doxorubicin (Figure 9B). Thus, cJun knockdown had differential effects on doxorubicin sensitivity depending on the concentrations of doxorubicin. 

**Figure 10:**
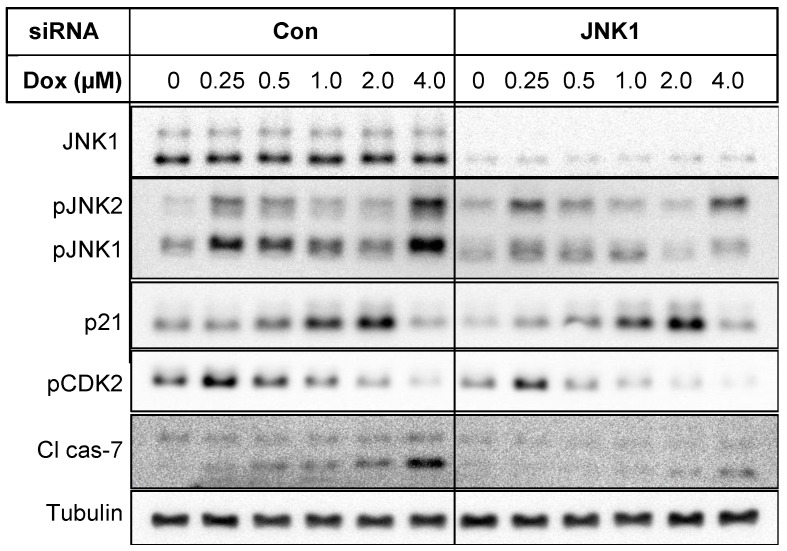
T47D cells were transfected with control non-targeting siRNA or JNK1 siRNA and then treated with indicated concentrations of doxorubicin. Western blot analyses were performed with indicated antibodies.

Since cJun is a downstream target of JNK, we determined the effects of doxorubicin on JNK phosphorylation. As shown in Figure 10, doxorubicin had a biphasic response on phosphorylated JNK1/2. Low concentrations of doxorubicin increased the phosphorylation of JNK1 and -2 (Figure 10) and total cJun (Figure 9) followed by a gradual decrease up to 2 µM doxorubicin. Treatment of cells with 4 µM doxorubicin caused an increase in phosphorylated JNKs (Figure 10) and increased processing of procaspase-7. Knockdown of JNK1 attenuated the increase in cleaved caspase-7, suggesting that activation of JNK at high concentrations of doxorubicin promotes apoptosis.

It has been reported that p21 is phosphorylated by CDK2 and phosphorylation of p21 alters its ability to function as an inhibitor of CDK2 [21]. The anticancer drug cisplatin was shown to induce p21 and phosphorylation of p21 by CDK2 contributed to cisplatin cytotoxicity even when p21 level was high [21]. We found that low concentrations of doxorubicin increased phosphorylation of CDK2 at the activating site, but higher concentrations of doxorubicin decreased rather than increased pCDK2 (Figure 10). Thus, an increase in CDK2 activity could not explain doxorubicin cytotoxicity. We, however, could detect an increase in the upper band of p21 with 1 and 2 µM doxorubicin. It is conceivable that phosphorylation of p21 by CDK2 inhibits its function as a cell cycle inhibitor contributing to the appearance of cleaved caspase-7 at low concentrations of doxorubicin.

We previously showed that S6K2 knockdown sensitized T47D cells to doxorubicin via JNK-mediated downregulation of the anti-apoptotic protein Mcl-1 [20]. Since high concentrations of doxorubicin caused activation of JNK (Figure 9B), we determined if Mcl-1 downregulation contributed to the increased sensitivity to doxorubicin caused by the combined knockdown of S6K2 and cJun. As shown in Figure 9B, S6K2 knockdown caused a substantial decrease in Mcl-1 and knockdown of both S6K2 and cJun further reduced Mcl-1 levels. These results suggest that the silencing of S6K2 increased sensitivity of T47D cells to doxorubicin by decreasing both the cell cycle inhibitor p21 and the pro-survival protein Mcl-1 in T47D cells. 

## 4. Discussion

Ribosomal S6 kinase 2 or S6K2, the gene product of *RPS6KB2*, is considered a neglected kinase since most of the earlier studies focused on its homolog p70S6K or S6K1 as the downstream target of mTORC1 [22]. S6K2 was originally discovered as a kinase that phosphorylates ribosomal protein S6 and shares functional similarity with its closely related homolog S6K1 [3,4,5]. Recent studies, however, suggest that these two homologs may exhibit distinct or even opposite functions [6], and interests in S6K2 are escalating. Since there is a paucity of information regarding the cellular targets of S6K2, we took an unbiased transcriptome profiling to identify potential downstream effectors of S6K2 and made a novel observation that silencing of S6K2 attenuated the expression of the cell cycle inhibitor p21 (*CDKN1A*). We validated our RNA-Seq data using both RT-qPCR and Western blot. Although p21 is a well-known target of p53, our study suggests that S6K2 also regulates p21 via the p53-independent pathway since the silencing of S6K2 decreased p21 expression in not only MCF-7 cells that contain wild-type p53 but also in T47D cells that contain mutant p53.

Both S6K homologs S6K1 and S6K2 have been associated with breast cancer, but the mechanisms by which they contribute to breast cancer may vary [6]. It was reported that S6K1 status is correlated with HER2-positive tumors whereas S6K2 status is correlated with ER-positive tumors [23]. p21 has also been implicated in ER-positive breast cancer. It has been reported that there is a positive correlation between ER and p21 in breast cancer cell lines and tumor specimens, and p21 plays an important role in the ER-signaling pathway [24,25,26,27]. We found that knockdown of S6K2 but not S6K1 decreased p21 in both MCF-7 and T47D cells. In fact, in some experiments, S6K1 knockdown appeared to increase p21 in MCF-7 cells, but based on several experiments the increase in p21 was not statistically significant. Although S6K1 knockdown caused a modest decrease in p21 in T47D cells, the effect of S6K1 knockdown on p21 level was much less compared to S6K2 knockdown.

Several studies suggest that S6K2 may regulate p21 via Akt. We have previously shown that S6K2 acts upstream of the Akt signaling pathway in MCF-7 cells [19]. Coexpression of pAkt and S6K2 was reported in the nucleus of breast cancer patients [23]. Several signaling pathways, including Akt, can also regulate p21 [28,29]. Therefore, we entertained the possibility that S6K2 regulates p21 via Akt. Since there are multiple Akt isoforms that may have distinct functions [29], we examined whether Akt1 or Akt2 mediates the effect of S6K2 on p21 levels. Our study suggests that Akt1 was primarily responsible for regulating p21. Knockdown of Akt1 decreased p21 whereas overexpression of Akt1 enhanced p21 levels in both MCF-7 and T47D cells. Akt1 overexpression partially rescued the effects of S6K2 knockdown in downregulating p21 in MCF-7 cells, but it had little effect in T47D cells, suggesting that S6K2 regulates p21 via the Akt1-dependent pathway in MCF-7 cells but the Akt1-independent pathway in T47D cells. It is conceivable that S6K2 also acts downstream of Akt in regulating p21 since S6K2 was identified as a nuclear target of Akt [4], and S6K2 is a downstream target of mTORC1, which acts downstream of Akt [30]. 

Since p21 is an inhibitor of cyclin-dependent kinases, it is counterintuitive that knockdown of S6K2, which has been associated with breast cancer survival, decreased p21 expression. However, p21 has been associated with both tumor promotion and tumor suppression [31]. p21 expression was shown to be elevated in several cancers, including breast cancer [32]. p21 can contribute to its oncogenic functions via several different mechanisms [32]. p21 is a substrate for Akt and phosphorylation of p21 results in its translocation from the nucleus to the cytoplasm, where it can exert its oncogenic function. It has been reported that Akt can cause an increase in p21 mRNA and protein expression as well as increased phosphorylation of p21 in the nucleus [33]. In addition, nuclear p21 has been associated with poor survival of patients with breast cancer [34]. 

We, and others, have shown that S6K2 protects against cell death by apoptotic stimuli [6,19,20,22,35]. Doxorubicin is a widely used chemotherapeutic drug used for the treatment of many cancers. It may induce cell proliferation, cell cycle arrest, and cell death depending on the concentrations and duration of exposure to doxorubicin and cellular context [36,37]. p21 was also shown to protect cells from stress or p53-dependent and p53-independent apoptosis [32,38]. In addition, resistance to chemotherapeutic drugs has been associated with the induction of p21 [28,39,40]. Moreover, *CDKN1A* mRNA was shown to be upregulated in glioblastoma cells and tissues and contributed to temozolomide resistance acting downstream of Akt [41]. A recent study reported that high levels of p21 conferred drug resistance in T47D cells [42]. 

It has been reported that in MCF-7 cells, low concentrations of doxorubicin upregulate p21 causing p53-dependent cell cycle arrest and DNA repair, but higher concentrations of doxorubicin induce proteosome-mediated downregulation of p21 causing induction of apoptosis [43]. We have previously shown that S6K2 promotes cell survival via Akt in MCF-7 cells [19] and the current study shows that knockdown of S6K2 decreases p21 via Akt. Thus, a Akt1-mediated decrease in p21 by S6K2 knockdown could partly explain the sensitization of MCF-7 cells to an apoptotic stimulus. 

Since T47D cells contain mutant p53, we primarily focused on T47D cells. In these cells, however, S6K2 knockdown decreased p21 via the Akt-independent pathway. Moreover, Akt1 knockdown did not enhance the sensitivity of T47D cells to doxorubicin. We have previously shown that silencing of S6K2 enhanced doxorubicin-induced apoptosis via activation of JNK1, independent of the Akt signaling pathway in T47D cells [20]. JNK is known to phosphorylate cJun, which binds to AP1 and enhances its transcriptional activity [44]. Based on our unbiased RNA-Seq analyses, we found that knockdown of S6K2 increased *JUN* mRNA, and Western blot analyses validated that S6K2 knockdown downregulates p21 via an increase in cJun. Since cJun binds to AP1 and enhances its transcriptional activity, it was unexpected that an increase in cJun would downregulate p21. Our results are, however, consistent with the report that cJun repressed p21 promoter activity [45]. In addition, downregulation of p21 by cJun sensitized KB-3 cells with compromised p53 function to vinblastine [46].

Our results suggest that S6K2 knockdown combats chemoresistance by decreasing drug-induced p21 levels since knockdown of either S6K2 or that p21 sensitized T47D cells to doxorubicin. Since S6K2 regulates p21 via cJun and the downregulation of cJun induces p21, an increase in cJun was expected to increase doxorubicin sensitivity. However, both p21 and cJun levels declined when cells were treated with doxorubicin concentrations that induced apoptosis, presumably due to the caspase-mediated cleavage and/or downregulation via the ubiquitin proteasome-mediated pathway [47,48]. 

cJun is a substrate for JNK, and we have shown that low levels of DNA damage caused by doxorubicin caused activation of the stress-activated protein kinase JNK. However, doxorubicin had a biphasic effect on JNK. A transient increase in pJNK was followed by its gradual decline, but higher concentrations caused an increase in JNK. It is well established that JNK has a dual role in cell survival and cell death [37,49]. While early or transient activation of JNK promotes cell survival, late or prolonged activation causes cell death [44,49]. We have shown that activation of JNK increased the processing of procaspase-7, and JNK1 knockdown attenuated doxorubicin-induced generation of cleaved caspase-7. We have previously shown that S6K2 knockdown sensitized T47D cells to doxorubicin via activation of JNK1, which promotes downregulation of the anti-apoptotic protein Mcl-1 [20]. It has also been reported that overexpression of p21 delays downregulation of Mcl-1 [50]. While it is not clear how cJun regulates Mcl-1, our results show that a decrease in Mcl-1 by combined knockdown of S6K2 and cJun was associated with increased sensitivity of T47D cells to doxorubicin. 

Since both cJun and JNK can function as pro- and anti-apoptotic proteins [37,49,51], it could be challenging to target these pathways. Our novel observations demonstrating that knockdown of S6K2 sensitized T47D cells containing mutant p53 to doxorubicin by downregulating the cell cycle inhibitor p21 as well as the anti-apoptotic protein Mcl-1 suggest that S6K2 could serve as an important target for combination chemotherapy. 

## 5. Conclusions

In summary, we have identified the cyclin-dependent kinase inhibitor p21/*CDKN1A* as a novel downstream target of S6K2 in ER-positive breast cancer cells. S6K2 was shown to regulate p21 via the cJun transcription factor independent of p53. S6K2 is known to function both downstream and upstream of the PI3K/Akt/mTOR pathway [6]; however, the contribution of Akt isoform was not known. We showed that S6K2 regulates p21 via Akt1 but p21 may also be regulated via the JNK signaling pathway. Furthermore, an increase in p21 by the chemotherapeutic drug doxorubicin could contribute to chemoresistance since a decrease in p21 sensitized cells to doxorubicin. Thus, p21 acts downstream of S6K2 to promote the survival of breast cancer cells. We propose that S6K2 knockdown enhances chemosensitivity via two distinct mechanisms: It combats drug resistance by lowering the drug-induced p21 levels, and enhances apoptosis by downregulating the anti-apoptotic protein Mcl-1.

## Data Availability

The data are available in the Supplementary Table.

## Supplementary Materials

The following supporting information can be downloaded at: https://www.mdpi.com/article/10.3390/cancers16223783/s1. Table S1: 118 significantly differentially expressed genes (DEGs) upon silencing of *RPS6KB2*; Table S2: Representative Gene Ontology terms of enriched functional clusters in shared 118 DEGs.
