# Supplementary material for: p21Waf1/Cip1 Is a Novel Downstream Target of 40S Ribosomal S6 Kinase 2
Source: Cancers (Basel). 2024 Nov 10;16(22):3783. doi: 10.3390/cancers16223783 (PMC11592183; doi:10.3390/cancers16223783)
Supplement: Supplementary file 1 [file cancers-16-03783-s001.zip › cancers-3303018-supplementary.pdf]

Supplementary Tables:

Supplementary Table S1: 118 significantly differentially expressed genes (DEGs) upon silencing of *RPS6KB2*.

GeneName	DESeq						Cuffdiff				
	Control	RPS6KB2_KD	log2FC	p-value	FDR		Control	RPS6KB2_KD	log2FC	p-value	FDR
**RPS6KB2**	2602.503	281.2957	-3.2097	4E-299	1.3E-294		96.0089	7.1258	-3.752	0.00005	0.00343
**PIP**	5118.926	1564.6764	-1.71	2.6E-154	3.7E-150		469.71	142.324	-1.7226	0.00005	0.00343
**B4GALT1**	10248.25	3881.7157	-1.4006	1.4E-129	1.4E-125		93.7419	36.2711	-1.3699	0.00005	0.00343
**CXCL16**	2421.217	680.771	-1.8305	1.6E-124	1.1E-120		65.0982	21.9531	-1.5682	0.00005	0.00343
**SIPA1L2**	562.3617	1808.2174	1.685	2.03E-93	1.17E-89		3.5682	11.2232	1.6532	0.00005	0.00343
**MYBL1**	2022.806	4820.7256	1.2529	2.36E-87	1.14E-83		19.6199	50.5711	1.366	0.00005	0.00343
**PRPSAP1**	2370.029	865.8472	-1.4527	1.34E-84	4.83E-81		89.3802	40.0535	-1.158	0.00005	0.00343
**CDKN1A**	2509.375	951.4602	-1.3991	1.09E-81	3.5E-78		46.9269	18.0491	-1.3785	0.00005	0.00343
**HIST1H2BK**	3180.722	1290.5412	-1.3014	1.37E-80	3.6E-77		180.361	72.5948	-1.313	0.00005	0.00343
**PEBP1**	8924.119	4250.8152	-1.07	2.67E-77	5.93E-74		259.958	124.04	-1.0675	0.00005	0.00343
**UAP1L1**	2014.186	4347.1336	1.1099	3.31E-68	5.62E-65		22.3679	47.6183	1.0901	0.00005	0.00343
**ACBD3**	3104.633	1419.5762	-1.129	1.26E-61	1.83E-58		28.9997	13.2496	-1.1301	0.00005	0.00343
**STOML2**	2423.536	1083.0629	-1.162	4.18E-58	4.83E-55		82.0261	37.6521	-1.1234	0.00005	0.00343
**MAN1A1**	539.1409	1406.3888	1.3833	1.33E-57	1.37E-54		3.9155	10.1241	1.3705	0.00005	0.00343
**SERPINI1**	702.7551	1658.6851	1.2389	6.45E-53	6.01E-50		28.4175	65.7006	1.2091	0.00005	0.00343
**STARD3**	1674.017	737.8372	-1.1819	5.95E-50	4.78E-47		80.781	35.9366	-1.1686	0.00005	0.00343
**KLF6**	1472.13	3050.1598	1.051	2.11E-49	1.65E-46		17.5899	39.7426	1.1759	0.00005	0.00343
**PTGES**	562.3388	155.9557	-1.8503	3.3E-47	2.38E-44		41.9546	11.2709	-1.8962	0.00005	0.00343
**JUN**	1006.156	2064.2028	1.0367	8.57E-44	5.39E-41		11.2752	22.9339	1.0243	0.00005	0.00343
**MANBAL**	1944.243	947.7859	-1.0366	1.11E-42	6.42E-40		63.3792	31.0371	-1.03	0.00005	0.00343
**ALDH3B2**	893.9717	347.2517	-1.3642	1.04E-41	5.67E-39		15.9915	6.6323	-1.2697	0.00005	0.00343
**SGK3**	479.3024	1118.7843	1.2229	1.1E-40	5.56E-38		18.9174	49.7162	1.394	0.00005	0.00343
**SIPA1**	930.1686	377.4476	-1.3012	1.63E-40	8.15E-38		34.7358	12.7573	-1.4451	0.00005	0.00343

**ALDH4A1**	1152.415	508.1598	-1.1813	6.71E-39	2.94E-36	25.7858	11.6376	-1.1478	0.00005	0.00343
**TCF19**	2356.834	1053.9959	-1.161	2.07E-38	8.21E-36	32.8675	14.4489	-1.1857	0.00005	0.00343
**TSC22D2**	680.5442	1431.4715	1.0727	3.31E-38	1.29E-35	8.5179	17.8419	1.0667	0.00005	0.00343
**LRRC8A**	1579.277	775.7907	-1.0255	1.38E-37	5.2E-35	14.9141	7.0834	-1.0742	0.00005	0.00343
**CTSD**	30459.46	12752.1793	-1.2561	1.23E-36	4.4E-34	992.078	419.965	-1.2402	0.00005	0.00343
**TK2**	639.6114	232.796	-1.4581	1.31E-36	4.64E-34	17.8712	5.8412	-1.6133	0.00075	0.0309
**ATP2A3**	1089.702	494.2137	-1.1407	1.04E-35	3.49E-33	11.1156	5.3855	-1.0454	0.0012	0.0439
**KRT15**	988.76	443.7597	-1.1558	1.55E-34	5.04E-32	25.9895	11.8736	-1.1302	0.00005	0.00343
**MYO1B**	816.439	1658.3363	1.0223	2.4E-34	7.7E-32	6.6957	14.9743	1.1612	0.00005	0.00343
**SNX33**	1061.445	488.4104	-1.1199	3.05E-34	9.68E-32	9.3836	4.1265	-1.1852	0.0006	0.0264
**FAM102B**	532.2626	1122.5583	1.0766	8.57E-33	2.64E-30	3.4436	7.2039	1.0648	0.00005	0.00343
**MARCH3**	224.075	588.0799	1.392	7.4E-32	2.18E-29	9.8713	23.4108	1.2459	0.00005	0.00343
**CYP26B1**	150.5769	450.56	1.5812	3.97E-31	1.14E-28	2.03	5.3684	1.403	0.0001	0.00634
**SCML1**	467.5173	988.6806	1.0805	1.58E-30	4.34E-28	6.8623	14.0446	1.0333	0.00005	0.00343
**TGFB2**	186.8044	488.6099	1.3872	3.34E-27	6.86E-25	3.0087	9.4787	1.6555	0.00005	0.00343
**PPP1R15A**	351.8902	760.5987	1.112	1.03E-26	2.01E-24	5.6602	12.1905	1.1068	0.00005	0.00343
**HMOX1**	257.4972	597.3192	1.2139	4.95E-26	8.88E-24	6.0815	13.2556	1.1241	0.00005	0.00343
**CPOX**	760.9666	361.9456	-1.0721	1.63E-25	2.82E-23	11.3198	5.098	-1.1508	0.0004	0.0194
**MET**	56.597	226.1095	1.9982	3.9E-24	5.99E-22	0.7508	2.9091	1.9541	0.0001	0.00634
**RRAGD**	192.8181	472.9513	1.2945	6.37E-24	9.65E-22	3.8033	9.8624	1.3747	0.00005	0.00343
**IDH1**	1379.232	663.2116	-1.0563	2.27E-23	3.28E-21	22.6945	11.3145	-1.0042	0.00005	0.00343
**NKAIN1**	329.6793	676.6126	1.0373	7.51E-22	9.57E-20	5.9941	12.024	1.0043	0.00005	0.00343
**DICER1**	2389.705	5655.1328	1.2427	1.09E-21	1.36E-19	22.6357	59.6252	1.3973	0.0008	0.0323
**RAB40B**	409.5788	166.2274	-1.301	1.51E-21	1.84E-19	14.7176	5.1769	-1.5074	0.00035	0.0175
**SHISA2**	104.2858	296.5091	1.5075	1.17E-20	1.3E-18	1.3719	3.8658	1.4946	0.00005	0.00343
**ZSWIM6**	256.5204	546.0637	1.09	1.94E-20	2.1E-18	1.6956	3.573	1.0754	0.00005	0.00343
**EPHX2**	579.5229	281.0962	-1.0438	4.43E-20	4.59E-18	14.3176	6.8327	-1.0673	0.00005	0.00343
**HMGCS2**	185.5522	47.7626	-1.9579	7.54E-20	7.68E-18	4.3121	1.0875	-1.9874	0.00005	0.00343
**INHBE**	54.5032	198.5877	1.8654	8.95E-20	9.01E-18	1.3051	4.538	1.7978	0.00005	0.00343

**TET1**	239.5301	486.1923	1.0213	7.4E-17	5.55E-15	0.9214	1.8533	1.0082	0.00005	0.00343
**HELZ2**	1067.185	2612.1506	1.2914	8.96E-17	6.61E-15	11.3843	30.9079	1.4409	0.00025	0.0134
**CREB5**	127.3867	308.7746	1.2773	9.19E-17	6.76E-15	1.5089	4.425	1.5522	0.00005	0.00343
**SLITRK6**	8.0183	76.997	3.2634	2.71E-16	1.92E-14	0.0681	0.6486	3.2521	0.00005	0.00343
**ELAVL2**	44.335	157.4011	1.8279	9.27E-16	6.15E-14	0.4399	2.0568	2.2253	0.00005	0.00343
**SAMD9**	119.8377	282.4955	1.2371	1.22E-14	7.23E-13	0.6437	1.5256	1.245	0.00005	0.00343
**FAM69A**	149.947	317.8713	1.084	2.27E-13	1.18E-11	2.2101	4.6419	1.0706	0.00005	0.00343
**MAP1B**	97.6983	229.8514	1.2343	1.19E-12	5.52E-11	0.2888	0.7399	1.3574	0.00035	0.0175
**GEM**	144.474	302.7149	1.0671	1.23E-12	5.67E-11	2.5905	8.29	1.6781	0.00005	0.00343
**FSCN1**	177.3809	425.2101	1.2613	1.69E-12	7.67E-11	3.6385	7.8177	1.1034	0.00035	0.0175
**RP11-328N19.1**	157.9244	319.4273	1.0163	3.7E-12	1.59E-10	4.2237	9.3808	1.1512	0.00005	0.00343
**MYCL**	178.1639	378.2878	1.0863	5.85E-12	2.46E-10	2.4362	5.124	1.0726	0.00005	0.00343
**ANGPT1**	83.7556	195.7323	1.2246	6.83E-11	2.55E-09	1.0162	2.5157	1.3078	0.00005	0.00343
**TNF**	11.4536	65.3367	2.5121	1.13E-10	4.12E-09	0.2742	1.54	2.4895	0.00005	0.00343
**EPHA4**	101.9981	229.4598	1.1697	1.26E-10	4.54E-09	1.4503	3.8376	1.4039	0.0007	0.0291
**IRF1**	94.7399	204.1313	1.1075	1.23E-09	3.82E-08	2.5382	6.233	1.2961	0.00005	0.00343
**PLSCR1**	481.7354	1092.4878	1.1813	2.08E-09	6.19E-08	18.7126	50.2397	1.4248	0.00005	0.00343
**LRRC26**	349.8142	163.7388	-1.0952	3.61E-09	1.03E-07	17.2621	8.0682	-1.0973	0.00005	0.00343
**ZSCAN12P1**	11.7366	59.4905	2.3416	3.96E-09	1.13E-07	0.2818	1.8662	2.7272	0.00005	0.00343
**SAMD9L**	67.1452	157.8926	1.2336	5.2E-09	1.44E-07	0.5035	1.4194	1.4953	0.00115	0.0423
**LUM**	54.6078	159.3842	1.5453	5.75E-09	1.57E-07	1.79	6.1688	1.785	0.00005	0.00343
**IRS2**	74.5973	168.086	1.172	5.95E-09	1.62E-07	0.393	0.8788	1.161	0.00005	0.00343
**LRRC31**	108.6672	40.9409	-1.4083	3.87E-08	9.12E-07	2.0611	0.8311	-1.3103	0.00005	0.00343
**IFI44**	10.8797	52.9145	2.282	6.01E-08	1.38E-06	0.4264	2.1514	2.335	0.00005	0.00343
**EIF4E1B**	153.0099	71.0048	-1.1076	9.11E-08	2.02E-06	6.6925	3.0629	-1.1276	0.0009	0.0352
**C1orf64**	146.3255	68.2277	-1.1008	2.7E-07	5.48E-06	1.483	0.6167	-1.2659	0.00005	0.00343
**LAMP3**	186.886	498.2624	1.4147	3.48E-07	6.88E-06	2.7533	8.1219	1.5607	0.00005	0.00343
**ZBTB18**	65.4799	138.7946	1.0838	6.98E-07	0.000013	0.6271	1.3181	1.0717	0.00005	0.00343
**PCYT1B**	35.5568	90.9323	1.3547	9.41E-07	1.71E-05	0.4754	1.445	1.6037	0.00005	0.00343

**ISG15**	118.7793	460.177	1.9539	1.56E-06	2.69E-05	8.1868	31.4735	1.9428	0.00005	0.00343
**TFF1**	84.9517	32.7306	-1.376	2.29E-06	3.82E-05	8.5526	3.2612	-1.391	0.00005	0.00343
**ANKRD1**	18.138	87.9737	2.2781	4.42E-06	6.96E-05	0.3574	1.7199	2.2667	0.00005	0.00343
**ZNF729**	49.1603	107.4988	1.1288	4.58E-06	7.18E-05	0.4891	1.0425	1.0919	0.00005	0.00343
**KLF15**	31.6369	84.9723	1.4254	1.13E-05	0.000164	0.4685	1.2912	1.4626	0.0001	0.00634
**RP11-363E7.4**	115.4001	55.8055	-1.0482	1.17E-05	0.000169	2.289	1.0957	-1.0628	0.00005	0.00343
**CASC1**	63.4268	23.0072	-1.463	1.19E-05	0.000171	2.458	0.695	-1.8224	0.00005	0.00343
**TXNDC5**	382.3003	172.7926	-1.1457	1.35E-05	0.000191	101.785	44.1234	-1.2059	0.00005	0.00343
**APOL1**	32.6953	78.8234	1.2695	1.43E-05	0.000202	0.5355	1.6232	1.5998	0.00005	0.00343
**KCNE4**	27.1993	69.7622	1.3589	1.79E-05	0.000246	0.3449	0.8989	1.3821	0.00005	0.00343
**CNTFR**	41.4659	116.7522	1.4935	1.97E-05	0.000268	0.8316	2.6897	1.6935	0.00005	0.00343
**EPHB2**	46.5819	99.4559	1.0943	1.97E-05	0.000268	0.5016	1.0405	1.0526	0.00135	0.0478
**CLEC7A**	44.4319	13.0382	-1.7689	2.36E-05	0.000316	2.2498	0.4527	-2.3132	0.0005	0.0231
**MX1**	935.9348	4084.8588	2.1258	2.54E-05	0.000337	15.1662	63.5612	2.0673	0.00005	0.00343
**IFI6**	476.0481	2283.6141	2.2621	8.46E-05	0.000988	26.3394	125.123	2.2481	0.00005	0.00343
**IFI27**	58.747	424.8118	2.8542	9.67E-05	0.00111	12.0633	58.3053	2.273	0.00005	0.00343
**SOX5**	20.571	64.4643	1.6479	9.84E-05	0.00113	0.3426	1.7281	2.3347	0.00005	0.00343
**DDX60**	130.2813	468.9463	1.8478	0.000111	0.00126	1.7123	4.9943	1.5444	0.00005	0.00343
**ATF3**	435.3703	920.0868	1.0795	0.000146	0.0016	14.262	30.2701	1.0857	0.00005	0.00343
**EHD3**	77.1272	35.7213	-1.1105	0.000169	0.00183	0.6755	0.3241	-1.0597	0.0001	0.00634
**KISS1R**	26.3909	61.26	1.2149	0.000244	0.00253	0.9422	2.1558	1.1941	0.0005	0.0231
**AKR1C3**	20.1426	50.9207	1.338	0.000311	0.00315	0.6676	1.7225	1.3674	0.00005	0.00343
**IFIT1**	287.7288	1489.8322	2.3724	0.00049	0.00472	5.9709	30.6819	2.3614	0.00005	0.00343
**SLC39A2**	53.9779	24.1608	-1.1597	0.000739	0.00676	1.6437	0.7389	-1.1535	0.00015	0.00886
**IL32**	19.4718	46.7088	1.2623	0.0012	0.0105	1.3746	2.8063	1.0297	0.0003	0.0154
**TMPRSS11E**	16.2303	40.2466	1.3102	0.00124	0.0107	0.2936	1.044	1.83	0.00005	0.00343
**OASL**	115.2956	272.2774	1.2397	0.00163	0.0136	2.2872	5.1774	1.1786	0.00005	0.00343
**IFITM1**	226.7605	545.2383	1.2657	0.00182	0.015	15.8494	38.6497	1.286	0.00005	0.00343
**RP11-391M1.4**	55.9416	26.6815	-1.0681	0.00192	0.0156	1.0187	0.4815	-1.0812	0.00005	0.00343

**ANKRD35**	39.1298	15.7155	-1.3161	0.00251	0.0198		0.499	0.1753	-1.509	0.0006	0.0264
**AGXT2**	31.54	5.9031	-2.4176	0.00336	0.0254		0.5259	0.0939	-2.4856	0.00005	0.00343
**LA16c-380H5.4**	50.4456	24.3744	-1.0494	0.00385	0.0286		6.1968	2.9398	-1.0758	0.00065	0.0278
**KRT8P45**	50.4941	25.0687	-1.0102	0.00475	0.0343		1.4189	0.6977	-1.0242	0.0008	0.0323
**RP11-566E18.3**	42.7181	19.3898	-1.1395	0.00503	0.036		0.5559	0.2275	-1.289	0.0001	0.00634
**KRT8P7**	32.3638	10.1934	-1.6667	0.00599	0.0418		0.9205	0.2867	-1.6826	0.00025	0.0134
**DNAH7**	30.02	11.9629	-1.3274	0.00601	0.0419		1.4035	0.2258	-2.6357	0.0005	0.0231
**XX-CR54.3**	36.9875	16.7908	-1.1394	0.00735	0.0494		1.6184	0.6981	-1.213	0.00005	0.00343

Supplementary Table S2: Representative Gene Ontology terms of enriched functional clusters in shared 118 DEGs.

Cluster	Enrichment Score	Top enriched GO terms in the cluster		
		**GO:term**	**FDR**	**gene list**
1	4.225	GO:0010033~response to organic substance	4.07E-04	PPP1R15A, CNTFR, **CDKN1A**, IFITM1, CPOX, IRS2, IFIT1, TNF, CXCL16, OASL, CYP26B1, CLEC7A, LAMP3, ANKRD1, HMOX1, MAN1A1, HMGCS2, EPHB2, CTSD, SOX5, EPHA4, TGFB2, JUN, ANGPT1, IDH1, MX1, AKR1C3, TET1, ISG15, SHISA2, DICER1, KLF15, PLSCR1, IFI27, IRF1, MAP1B, RRAGD, TFF1, ATF3
2	3.245	GO:0012501~programmed cell death	1.91E-03	PPP1R15A, CNTFR, **CDKN1A**, B4GALT1, ATP2A3, IFI6, IRS2, TNF, CYP26B1, CLEC7A, LAMP3, ANKRD1, HMOX1, CTSD, TXNDC5, TGFB2, JUN, ANGPT1, MX1, AKR1C3, DICER1, PLSCR1, IFI27, IRF1, PIP, SGK3, MET, ATF3
3	2.999	GO:1903901~negative regulation of viral life cycle	2.90E-04	IL32, IFITM1, JUN, PLSCR1, MX1, ISG15, IFIT1, TNF, OASL
4	2.157	GO:0030855~epithelial cell differentiation	1.70E-02	**CDKN1A**, JUN, B4GALT1, AKR1C3, KLF15, TNF, MYCL, CYP26B1, MAP1B, KRT15, SLITRK6, SLC39A2, MET, SIPA1
5	1.935	GO:1901700~response to oxygen-containing compound	4.16E-02	EPHA4, **CDKN1A**, TGFB2, JUN, AKR1C3, IRS2, KLF15, TNF, CYP26B1, CLEC7A, MAP1B, RRAGD, ANKRD1, HMOX1, TFF1, MAN1A1, HMGCS2, EPHB2, CTSD, MET, SIPA1
6	1.906	GO:0046685~response to arsenic-containing substance	4.97E-02	**CDKN1A**, HMOX1, CPOX, ATF3
7	1.809	GO:0051336~regulation of hydrolase activity	1.96E-02	PPP1R15A, EPHA4, TGFB2, ATP2A3, IFI6, SIPA1L2, IFIT1, TNF, OASL, PLSCR1, CLEC7A, LAMP3, SERPINI1, CTSD, MET, SIPA1
8	1.486	GO:0009893~positive regulation of metabolic process	6.49E-03	PPP1R15A, **CDKN1A**, STOML2, ATP2A3, IRS2, SNX33, TNF, HELZ2, CYP26B1, ZNF729, CLEC7A, LAMP3, ANKRD1, HMOX1, EPHB2, CTSD, MYBL1, IL32, ZBTB18, EPHA4, TGFB2, JUN, ANGPT1, AGXT2, LUM, EPHX2, AKR1C3, TET1, KLF15, INHBE, KLF6, PLSCR1, IRF1, PIP, RPS6KB2, MET, ATF3, CREB5
